# Quantifying the Relative Contributions of Exposure Time, ECG, GSR, and Vibration-Derived Features for Motion Sickness Prediction

**DOI:** 10.3390/s26154774

**Published:** 2026-07-27

**Authors:** Abeeb Opeyemi Alabi, Do-Kyung Kwak, Byoung-Gyu Song, Namcheol Kang

**Affiliations:** 1 Department of Mechanical Engineering, Kyungpook National University, Daegu 41566, Republic of Korea; alabiabeeb14@gmail.com (A.O.A.);; 2 Cognitive Science Research Group, Korea Brain Research Institute (KBRI), Daegu 41062, Republic of Korea; bgsong137@kbri.re.kr; 3 School of Mechanical Engineering, Kyungpook National University, Daegu 41566, Republic of Korea

**Keywords:** motion sickness, exposure time, electrocardiography, galvanic skin response, head acceleration, feature importance, whole-body vibration, machine learning

## Abstract

Motion sickness is expected to become more prevalent in road transportation as passengers increasingly engage in non-driving-related activities under automated driving. This study quantified the relative contributions of exposure time (discrete trial index), physiological signals, and vibration-derived features to motion sickness prediction under controlled road vibration. Thirty-nine participants were exposed to four road profiles reproduced on a motion simulator while electrocardiography (ECG), galvanic skin response (GSR), triaxial head acceleration, and motion sickness ratings were recorded across ten 2 min trials per session during a reading task. Three dataset configurations were compared: exposure time only (type 1), physiological and vibration-derived features only (type 2), and the combined (type 3) dataset. Motion sickness prediction was evaluated using support vector classification, random forest, and XGBoost, and permutation importance was used to assess feature contribution. Across all classifiers, exposure time was ranked as the most important feature, followed by overall root-mean-square, while ECG and GSR contributed at lower levels. The combined dataset achieved the best performance, reaching 77% classification accuracy and R^2^ values up to 0.49. Leave-one-subject-out (LOSO) cross-validation further confirmed the feature importance ranking under subject-level evaluation, while physiological features showed near-zero importance for unseen participants, suggesting their contributions are largely individual-specific.

## 1. Introduction

Motion sickness has become an increasingly important topic in motion environments such as vehicles, virtual reality systems, and simulators, due to its role in passenger discomfort. Sensory conflict theory describes motion sickness as a consequence of a mismatch between the motion predicted by the brain and the actual motion perceived through the sensory organs [1,2,3]. With the rapid evolution of mobility technologies, particularly the emergence of autonomous vehicles and future mobility systems, the likelihood of motion sickness will further increase. In these automated environments, passengers are afforded more passive travel time to engage in non-driving-related activities (NDRA), which can reduce their postural engagement and motion anticipation, and intensify sensory conflict among visual, vestibular, and somatosensory inputs [4]. Moreover, reliable detection and prediction of motion sickness remain challenging because symptom development evolves gradually, varies strongly across individuals, and is shaped by a complex combination of motion type or magnitude, duration of exposure, and accumulation of sensory conflict over time. Hence, there is an increasing need to understand the underlying mechanisms contributing to motion sickness.

In experimental and applied settings, motion sickness severity is still primarily assessed using subjective rating scales such as the misery scale (MISC) [5]. While these ratings convey perceived discomfort, they are vulnerable to expectancy effects, reporting bias, and limited temporal resolution for tracking continuous symptom progression during prolonged exposure [6]. Objective physiological signals have therefore received growing attention as complements or alternatives to subjective ratings over the past decade. Prior studies have investigated electroencephalography (EEG), electrocardiography (ECG), galvanic skin response (GSR), respiration, skin temperature, and head acceleration for motion sickness assessment [6,7,8,9]. EEG has been the most widely employed signal due to its direct representation of neural activity, while ECG and GSR features are explored for their ability to reflect sympathetic and parasympathetic autonomic nervous responses. Additionally, machine learning techniques have been applied to integrate physiological signals in a multimodal framework to improve motion sickness prediction performance [10,11,12]. These studies show that motion sickness is accompanied by measurable autonomic and neural changes that can be predicted and quantified through systematic feature engineering, multimodal fusion, and deep learning.

An important but insufficiently resolved issue in physiological motion sickness prediction is the relative contribution of cumulative exposure duration. Motion sickness severity is known to increase with both vibration magnitude and exposure duration [2,3,13]. Motion sickness dose value (MSDV), defined in ISO 2631-1 and widely adopted in vibration research, quantifies the cumulative effect of motion exposure by integrating frequency-weighted acceleration over time [14]. Critically, MSDV increases with exposure time under fixed, controlled and sustained vibration, making exposure time and MSDV functionally collinear. Despite this collinearity, prior studies, including machine learning models that integrate physiological signals alongside MSDV and other temporal features, have rarely decomposed the relative contribution of exposure time against autonomic responses [15,16]. Whether motion sickness model prediction is primarily driven by the temporal accumulation of exposure, by autonomic physiological responses to the exposure, or by the magnitude of head acceleration associated with the vibration input remains an open and practically important question.

Interpretability is another limitation of existing motion sickness prediction models. Several deep learning models integrated with physiological signals, especially EEG, have achieved high classification accuracies but provide limited insight into feature contributions or the specific physiological or biomechanical mechanisms driving motion sickness detection [15,16,17,18]. For instance, in our recent study [17], we showed that MSDV-weighted EEG segments can substantially improve cross-subject classification. While the effectiveness of MSDV as an EEG weighting factor was confirmed, there was no direct quantification of the predictive contribution of MSDV as a standalone or combined input feature, nor a disentanglement of the relative roles of temporal exposure progression, physiological responses, and vibration-based features in the improvement of prediction accuracy. Interpretable feature contribution methods such as permutation importance, local interpretable model-agnostic explanations (LIME), and Shapley additive explanations (SHAP) can provide model-agnostic quantification of feature contribution across multiple classifiers enabling access into their black-box predictions [19,20]. However, interpretability is limited in existing motion sickness prediction models, thereby hindering the development of efficient, low-cost, and deployable motion sickness monitoring systems. Such interpretable systems can help to ascertain whether simpler features such as exposure time and vibration metrics may suffice for practical applications, or whether physiological measurements are always necessary.

This study aims to address these knowledge gaps by systematically evaluating and quantifying the relative contributions of multimodal features in motion sickness prediction. Specifically, we analyze features derived from ECG, GSR, and triaxial acceleration signals, alongside exposure time, operationalized as a discrete trial index (1–10) and used as a proxy for cumulative exposure order, within each fixed vibration condition. To do so, we recorded ECG, GSR, and triaxial head acceleration from 39 subjects during vibration exposure to four road vibration profiles (smooth, rough, severely rough, and a hump road) reproduced on a motion simulator. Three dataset configurations were constructed: exposure time only (type 1), physiological and vibration-derived features only (type 2), and the combined (type 3) dataset integrating both. This design makes it possible to determine whether cumulative exposure time is merely a supportive variable or a dominant explanatory factor in motion sickness prediction. Classification and regression models were trained using support vector machines, random forest, and XGBoost, and permutation-based feature importance analysis was applied to quantify feature contributions across all predictive models. The specific aims of this study are: (i) to determine the extent to which exposure time (discrete trial index) alone explains motion sickness compared to physiological and vibration-derived features; (ii) to identify which individual features contribute most to motion sickness predictions when exposure time is excluded; and (iii) to derive practical guidelines for feature selection in motion sickness prediction that prioritize the most robust predictors while reducing sensor burden and model complexity. By prioritizing feature-level interpretability, this study aims to move beyond prediction accuracy alone and identify the features that matter most for efficient, scalable, and deployable motion sickness prediction systems.

## 2. Materials and Methods

### 2.1. Study Design and Participants

This study used a within-subject design to quantify the relative predictive value of exposure time, physiological responses, and vibration-based features for motion sickness prediction under controlled road-vibration exposure. A total of 42 participants (24 males and 18 females) were recruited, with a mean age of 22.90 ± 2.28 years. Inclusion criteria were no history of cardiovascular disease, normal or corrected-to-normal vision, and a self-reported history of motion sickness during car travel. Participants were instructed to abstain from alcohol the day before the experiment and refrain from smoking on the day of testing. All participants had lunch before the session. The Institutional Review Board of Kyungpook National University (approval no. 2023-0628) approved the study, and written informed consent was obtained from all participants prior to testing after a full explanation of the experimental procedures.

### 2.2. Experimental Protocol

**Figure 1:**
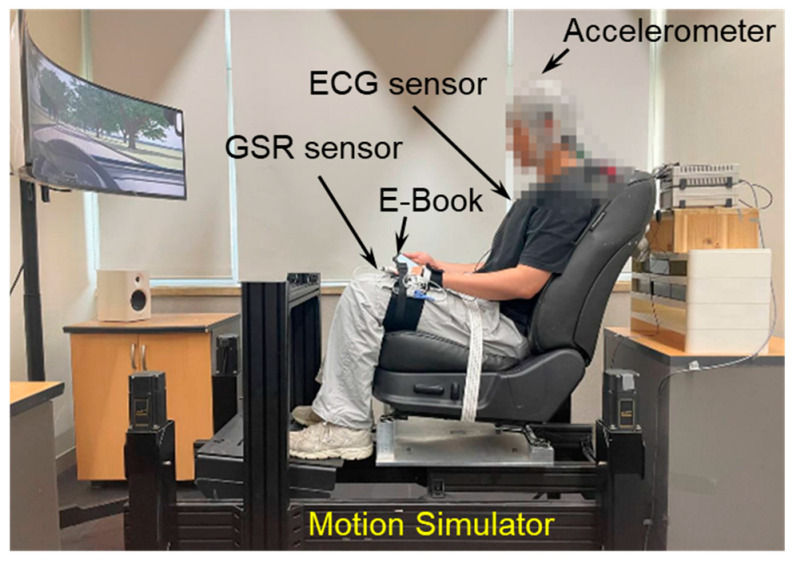
Experimental setup for whole-body vibration exposure using a motion simulator. Participants were seated on the simulator and performed NDRA by reading an e-book while ECG, GSR, and head acceleration were recorded using an accelerometer mounted on the head.

Whole-body vibration was induced using a motion simulator that reproduced controlled road-vibration conditions while ECG, GSR, and head acceleration were simultaneously recorded (Figure 1).

Participants read an e-book during vibration exposure to simulate non-driving-related activity (NDRA) conditions. The protocol included four road-vibration profiles: smooth (ISO 8608 class B), rough (ISO 8608 class D), severely rough (ISO 8608 class E) [21], and a hump road with regularly spaced parabolic speed bumps. Explicit details about each road conditions can be found in our previous work on this study [17]. These conditions were selected to create varying vibration intensities and examine how motion sickness evolves across different motion characteristics.

**Figure 2:**
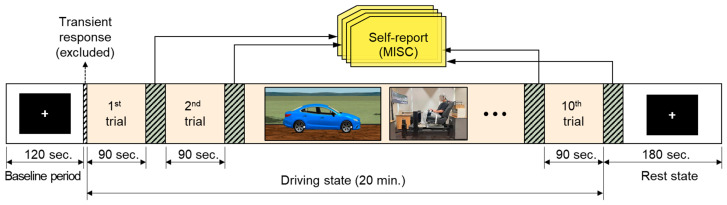
Experimental timeline within each road condition. A 2 min baseline period with fixation on a white cross preceded the exposure sequence. Each road profile lasted 20 min and was divided into ten consecutive 2 min trials. Each trial comprised a 90 s reading period followed by a 30 s response period for MISC reporting on the screen. A 3 min washout period was provided between road conditions.

Each participant completed four sessions, one per road profile, with session order randomized to minimize sequence effects. Each session began with a 2 min baseline period, followed by a 20 min exposure period and a 3 min washout period (Figure 2). The 20 min exposure period consisted of ten consecutive 2 min trials. Within each trial, participants read for 90 s and then paused to rate their motion sickness se-verity using the misery scale (MISC) via a screen-displayed questionnaire (Table 1). Each verbal rating took approximately 30 s, and data recorded during this period were excluded from analysis. The initial transient response immediately after the baseline period was also excluded, as it was used only for synchronization across the recorded signals. After each session, the washout period was extended until the participant’s MISC score returned to 0. If a participant reported severe nausea or requested to stop, the experiment was terminated immediately. No participant exceeded a MISC score of 9 or requested to stop, and all participants completed 40 trials across the four sessions.

### 2.3. Signal Acquisition and Synchronization

ECG, GSR, and head acceleration signals were recorded simultaneously throughout the experiment. ECG and GSR were acquired using the AIM Gen2 system (Cognionics, Inc., San Diego, CA, USA) at a sampling frequency of 500 Hz. ECG electrodes were attached to the infraclavicular regions to collect bipolar cardiac activity, and GSR electrodes were placed on the second phalanx of the left middle and ring fingers, with the ground electrode attached to the ulnar pulse point of the same wrist. Head acceleration was measured using a 3D accelerometer (Brain Products GmbH, Gilching, Germany) mounted on the participant’s cap, capturing linear acceleration along the x-axis (anterior–posterior), y-axis (left–right), and z-axis (superior–inferior). These acceleration signals were used to derive vibration-related features that reflect motion exposure and head-motion response.

Temporal alignment was performed to enable multimodal analysis of the recorded signals. Because hardware-level synchronization between the AIM Gen2 system and the accelerometer was not available, event-based synchronization was applied. At the onset of each road condition, we applied a mechanical impulse, which produced a clearly identifiable transient peak in the z-axis of the acceleration signal. Simultaneously, a characteristic deflection in the dedicated synchronization channel of the AIM2 system was applied to generate a clear peak in the physiological signals. These co-occurring peaks served as markers to align all sensor streams to a common temporal reference.

All recordings underwent quality screening before analysis. Trials with substantial measurement errors or signal artifacts were excluded. Data from three participants were fully excluded because of poor ECG or GSR quality, and one additional participant was partially excluded for one road condition that did not meet the quality criteria. There-fore, the final dataset used for analysis included 39 participants, with one partial exclusion.

### 2.4. Signal Processing and Feature Extraction

The recorded signals were designed to capture three complementary sources of information relevant to motion sickness prediction: temporal exposure, autonomic physiological response, and vibration intensity. ECG features were used to assess cardiac and autonomic responses, GSR to reflect sympathetic arousal, and acceleration signals to quantify vibration effects accumulated over the exposure period. Features were extracted on a per-trial basis using the 90 s reading window of each 2 min trial during the exposure period.

**Figure 3:**
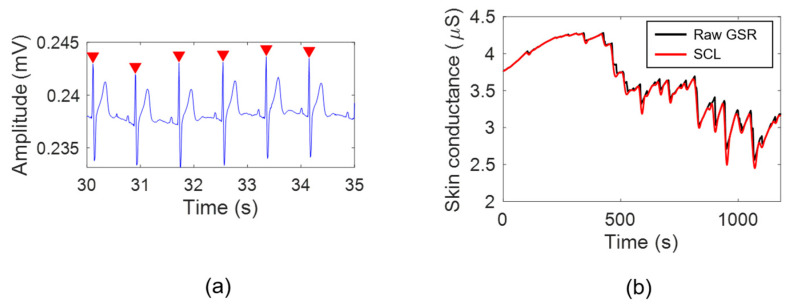
Signal processing for feature extraction from ECG and GSR. (**a**) ECG R-peak detection using the Hilbert-transform-based method, with detected R peaks marked on the raw ECG waveform. (**b**) Decomposition of the raw GSR signal into SCL using the cvxEDA algorithm.

ECG signals were processed using the Hilbert-transform method proposed in [22]. This method aligns R-peaks with positive zero crossings and verifies candidate peaks in the Hilbert-transformed signal by selecting the local maximum in the raw ECG. It is well suited for noisy vibration recordings and reduces dependence on aggressive preprocessing filters that could attenuate both vibration-induced artifacts and true cardiac signal components, enabling stable R-peak detection as shown in Figure 3a. Extracted ECG features included mean heart rate (HR) and mean RR-interval as cardiovascular indices representing overall cardiac activity; the root mean square of successive differences (RMSSD) and the percentage of successive normal-to-normal (NN) intervals differing by more than 50 ms (pNN50) as time-domain heart rate variability (HRV) indices representing short-term beat-to-beat variability; and normalized low-frequency power (LF_norm_) as a frequency-domain index related to autonomic modulation [23].

GSR signals were decomposed into tonic and phasic components using the cvxEDA algorithm [24]. The algorithm separates slow-varying skin conductance level (SCL) activity from faster skin conductance response (SCR) components. Because vibration exposure can introduce artifacts in SCR, the present analysis focused on SCL, which is more stable and better suited to short-term motion sickness monitoring. Figure 3b shows a sample raw GSR signal and the decomposed SCL signal. Two GSR features were extracted from the SCL component: mean SCL and SCL standard deviation. These features were intended to reflect tonic sympathetic arousal and within-trial variability.

Acceleration features were derived from the head acceleration signals using ISO 2631-based vibration metrics [13]. By applying a frequency-weighting function to the head acceleration signals, we computed axis-specific frequency-weighted root mean square (RMS) acceleration in the x-, y-, z- axes, and overall RMS, as well as motion sickness dose value (MSDV) for each axis. These features represent both the instantaneous magnitude of vibration and the cumulative effects of continued exposure. MSDV was computed for each trial segment from the frequency-weighted acceleration signal and then accumulated across successive trials to represent cumulative exposure progression under the fixed vibration profiles used in this study.

### 2.5. Baseline Correction and Normalization

To account for inter-individual variability in physiological levels, all extracted biosignal features were baseline-corrected and normalized. Because physiological responses differ substantially across individuals in both baseline level and response amplitude, all extracted features were referenced to each participant’s baseline period. For each trial, the baseline-corrected feature value was expressed as the change from baseline:

(1) $$\Delta feature=feature_{trial}-feature_{baseline},$$

while normalized low-frequency power, LFnorm, was computed as follows:

(2) $$LF_{norm}=\frac{LF\;Power}{Total\;Power-VLF\;Power}\times 100,$$

expressed relative to the total spectral power excluding very-low-frequency (VLF) activity. For GSR, baseline normalization was performed using min–max scaling based on each participant’s baseline range:

(3) $${SCL}_{norm}\left(i\right)=\frac{SCL_{trial}\left(i\right)-\min\left(SCL_{baseline}\right)}{\max\left(SCL_{baseline}\right)-\min\left(SCL_{baseline}\right)},$$

Overall, baseline referencing and normalization were applied to reduce inter-subject noise and emphasize motion-induced changes rather than absolute physiological differences.

### 2.6. Feature Selection and Redundancy Analysis

**Figure 4:**
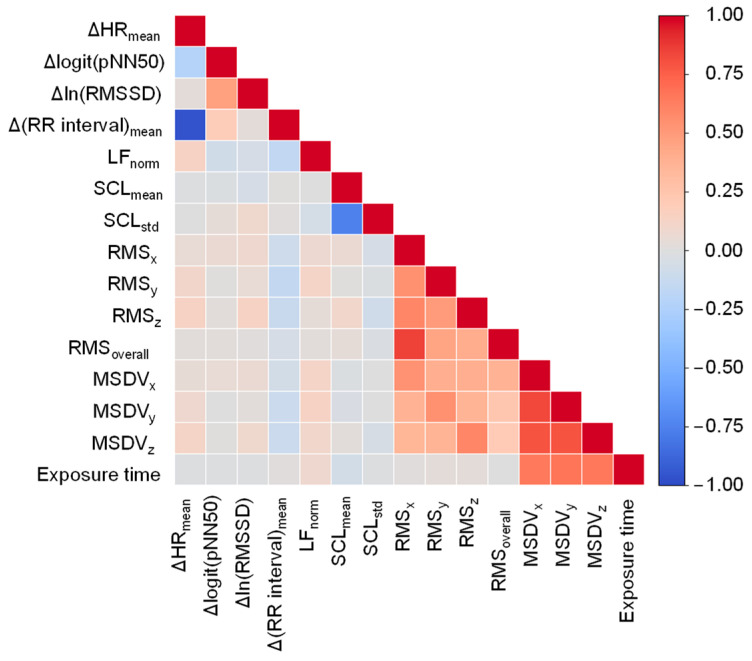
Pairwise correlation matrix of extracted features used for correlation-based feature selection. Features with absolute correlation coefficients above 0.5 were treated as redundant, and a single representative feature was retained for each highly correlated feature group to support stable permutation importance interpretation.

To reduce feature redundancy and improve the reliability of permutation importance analysis, a correlation-based feature reduction step was applied before interpretable model training. A total of 15 features, including exposure time, were extracted from the processed ECG, GSR, and acceleration signals. Because some features were strongly correlated, inter-feature correlation was examined using pairwise correlation analysis across all extracted features (Figure 4). 

A correlation threshold of |r| > 0.5 was used to remove redundant features conservatively and to preserve a compact, interpretable set of non-overlapping predictors for permutation-based importance analysis. When two features exceeded this threshold, only one representative feature was kept based on physiological interpretability and relevance to motion sickness mechanisms, in order to avoid destabilizing permutation importance where feature importance would be distributed across overlapping predictors. Mean change in heart rate (∆HR_mean_) and mean change in RR interval, (∆(RR interval)_mean_) were highly correlated; therefore, ∆(RR interval)_mean_ was retained because it is more directly related to cardiac pacing. Mean SCL (SCL_mean_) and SCL standard deviation (SCL_std_) were also highly correlated; as a result, SCL_mean_ was retained because it provides a clearer index of tonic sympathetic arousal. The three axis-specific RMS acceleration measures were mutually correlated; therefore, RMS_overall_ was retained because it captures overall motion magnitude across axes. Critically, all three axis-specific MSDV values were strongly correlated with exposure time, suggesting that under the fixed vibration profiles used in this study, MSDV and cumulative trial index were functionally collinear. As such, exposure time, operationalized as a discrete trial index (1–10), was retained as the representative temporal feature because it directly encodes cumulative exposure progression.

After feature selection and redundancy analysis, a set of seven features was retained for interpretable model training: ∆(RR interval)_mean_, ∆logit(pNN50), ∆ln(RMSSD), LF_norm_, SCL_mean_, RMS_overall_, and exposure time. These selected features provide a compact representation of temporal progression, physiological responses, and vibration-derived characteristics for motion sickness analysis.

### 2.7. Dataset Configurations for Feature Importance Analysis

Three dataset configurations were constructed to systematically compare the incremental contribution of each feature group in motion sickness prediction. Each dataset contained feature values extracted from individual trials and the corresponding MISC score.

The exposure time only (type 1) dataset contains exposure time as the sole feature. Exposure time was operationalized as a discrete trial index (1–10), which increased monotonically across the 2 min exposure intervals within each road condition. The type 1 dataset was used to test whether cumulative motion exposure alone could predict motion sickness progression.

The physiological and vibration-derived only (type 2) dataset combines ECG-, GSR-, and acceleration-derived features such as ∆(RR interval)_mean_, ∆logit(pNN50), ∆ln(RMSSD), LF_norm_, SCL_mean_, and RMS_overall_ without exposure time. This configuration represents the predictive capability of autonomic and motion-related signals alone and reflects the type of feature set commonly used in prior motion sickness studies.

The combined (type 3) dataset integrates all seven features, combining exposure time with physiological and vibration-derived features. This configuration was designed to evaluate whether adding temporal progression provides complementary information beyond autonomic and vibration-related responses.

Comparing these three datasets allowed us to determine whether exposure time alone acts as a dominant predictive feature, whether physiological and vibration features provide additional value, and which feature combination is most suitable for practical motion sickness prediction.

### 2.8. Statistical Analysis

Several statistical analyses were applied to examine how physiological responses varied with motion sickness severity and accumulated exposure time.

A group-level feature comparison was performed to assess changes across symptom severity categories. All trials were divided into two groups based on MISC score: weak motion sickness (MISC < 4) and strong motion sickness (MISC ≥ 4). The threshold of MISC ≥ 4 was selected because a MISC score of 4 corresponds to “fairly” perceptible discomfort on the scale’s symptom anchor, representing a qualitative transition from ambiguous or slight symptoms (scores 1–3) to clearly established motion sickness with fairly perceptible multisymptomatic experience. Between-group differences were evaluated using Welch’s unequal-variance *t*-test after the Shapiro–Wilk normality test indicated non-normal distributions. Mann–Whitney U tests were also performed as a nonparametric robustness check and showed consistent significance patterns. Cohen’s d effect sizes were calculated to quantify the magnitude of between-group differences. Univariate regressions of MISC scores against individual features provided coefficients of determination as an index of each feature’s severity explanatory strength.

Two averaging analyses were also conducted to examine how individual features varied with symptom severity and exposure time. First, in the severity-level averaging analysis, feature values were averaged within each discrete MISC score level across participants. Linear regression of the MISC-level averaged feature values against MISC scores revealed severity trends across participants. Second, in the exposure-time averaging analysis, feature values were averaged across the ten 2 min exposure intervals to characterize temporal feature dynamics under cumulative exposure. Linear regression of the exposure-time-averaged feature values against trial index quantified temporal accumulation effects. Direct comparison of these two analyses allowed us to determine whether each feature was more strongly associated with symptom severity progression, exposure time, or both. All computed statistics are included in the results section.

### 2.9. Machine Learning Framework and Feature Importance

Machine learning models were used as analytical tools to compare feature sets and assess the robustness of extracted feature importance patterns.

The emphasis of this study was not the development of a new classifier, but the identification of which feature groups were most informative for motion sickness prediction under different dataset configurations. Therefore, support vector classifiers (SVC) with a radial basis function kernel, random forest classifiers, and XGBoost classifiers were implemented in both classification and regression paradigms. These three algorithms represent complementary inductive biases, with SVC relying on margin maximization, random forest on ensemble averaging of decision trees, and XGBoost on gradient-boosted tree ensembles [25,26,27]. Evaluating feature importance patterns across these fundamentally different machine-learning mechanisms enabled assessment of the robustness and reliability of feature interpretability.

Classification was treated as the primary modeling framework because it directly reflects the practical task of distinguishing low and high motion sickness states and facilitates interpretation of feature contributions using performance-based metrics. Classification models were applied to distinguish the weak and strong motion sickness groups defined in Section 2.8. The weak MS (MISC < 4) = 877 trials and strong MS (MISC ≥ 4) = 673 trials, so we applied synthetic minority oversampling technique (SMOTE) to generate synthetic samples by interpolating within the feature space of the minority class. To preserve the natural class distribution during evaluation and prevent data leakage, subject-based partitioning was performed before oversampling, and SMOTE was applied only to the training fold within each cross-validation iteration. No synthetic samples were generated from the held-out data, and the original test folds were retained for evaluation. Regression models were additionally constructed to verify whether the observed feature contribution pattern remained consistent when MISC severity was treated as a continuous outcome rather than a binary label. The same model families and feature sets were used for regression. All models were trained and evaluated using stratified 10-fold cross-validation. In each fold, nine folds were used for training and one as the held-out test set. For classification, performance was reported using accuracy, area under the curve (AUC), precision, sensitivity, specificity, and F1 score. For regression, coefficient of determination (R^2^), mean squared error (MSE), and root mean squared error (RMSE) were reported.

To quantify feature contributions, permutation importance analysis was applied to the trained classification models. For each feature, values in the test fold were randomly permuted while all other features were held constant, and the resulting decrease in F1 score was recorded. This process was repeated 30 times per feature, and the mean decrease in F1 score was used as the final importance estimate. Importance values were normalized to percentage contributions to enable direct cross-model comparison. Permutation importance was computed for all classification models, and consistency of importance patterns across models was used to assess the robustness of feature contributions.

To evaluate generalization to unseen participants, leave-one-subject-out (LOSO) cross-validation was additionally performed on the type 2 and type 3 dataset configurations across all three classifiers, with one participant held out per fold (*n* = 39 folds). Permutation importance was also recalculated under LOSO using F1 score as the performance metric.

## 3. Results

### 3.1. Descriptive Summary of Motion Sickness Responses

A total of 1550 responses were collected during the experimental period, and the number of responses for each MISC score is shown in Table 2. The mean MISC score across all participants was 3.33 ± 0.05 (standard error), indicating that symptom severity generally remained in the lower-to-moderate range but increased progressively during exposure. Among the individual symptoms, dizziness was reported most frequently, followed by stomach awareness and headache. The response counts for dizziness, stomach awareness, and headache differed by fewer than three counts, indicating that no single symptom dominated the overall motion sickness experience, which is consistent with the multisymptomatic profile of vibration-induced motion sickness reported in previous road-vehicle studies [6]. A comparison of mean MISC scores between male and female participants showed no significant difference (*p* > 0.05), indicating that motion sickness susceptibility in this experiment was not gender-related.

### 3.2. Feature Differences Between Weak and Strong Motion Sickness

Statistical results for the group-level feature comparison between the weak and strong motion sickness groups are summarized in Table 3. Significant differences were observed for exposure time, LF_norm_, ΔHR_mean_, ∆(RR interval)_mean_, all axis-specific RMS measures, RMS_overall_, and MSDV features (*p* < 0.001, d > 0.4 in all cases). Among these, axis-specific MSDV features showed the largest effect sizes, suggesting that cumulative vibration exposure was strongly associated with higher motion sickness severity. ECG-derived features showed a mixed pattern: mean heart rate and RR interval showed significant group differences, whereas short-term HRV features such as RMSSD and pNN50 did not. LF_norm_ produced the largest effect size among the ECG features (d = 0.42), indicating that frequency-domain cardiac modulation was more informative than time-domain variability measures in this dataset. GSR features showed a more limited but still interpretable trend. Neither SCL_mean_ nor SCL_std_ showed significant group differences, although SCL_mean_ was higher in the strong motion sickness group than in the weak group. In addition, sample-level regression between MISC scores and individual features generally produced coefficients of determination below 0.1, except for exposure time and axis-specific MSDVs, which showed slightly higher values.

Overall, the statistical results, especially for the axis-specific MSDV features, with Cohen’s d ranging from 0.99 to 1.1, suggest that cumulative vibration exposure is the most discriminating signal between weak and strong motion sickness groups, while d = 0.81 for exposure time further strengthens the practicality of trial index as an experimental surrogate for MSDV, as designed in this study, and shows that both cumulative vibration exposure and temporal exposure order are more directly aligned with motion sickness progression than most physiological variables considered individually.

### 3.3. Trends Across Motion Sickness Severity and Exposure Time

**Figure 5:**
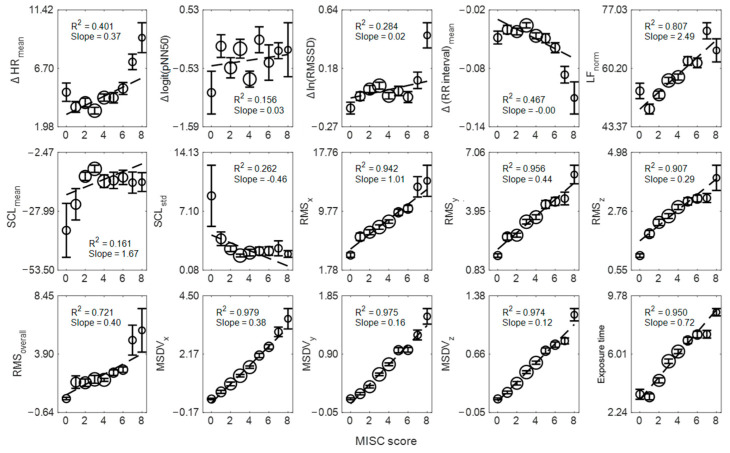
Severity-level averaged feature trends across MISC scores. Feature values were averaged within each MISC score level across participants, and error bars indicate standard error. Linear regression lines are shown for each feature, with regression slope and coefficient of determination reported in each panel.

The severity-level averaging analysis revealed strong linear associations between MISC scores and severity-level averaged feature values (Figure 5). In particular, LF_norm_ (R^2^ = 0.807), axis-specific RMS (R^2^ = 0.942–0.956), and axis-specific MSDV (R^2^ = 0.975–0.979) showed clear monotonic trends across MISC levels, indicating that these variables increased or changed systematically as symptoms became more severe. HR_mean_ and mean interval also showed moderate associations (R^2^ = 0.401 and 0.467). In contrast, pNN50, RMSSD, SCL_mean_, and SCL_std_ showed weak linear associations with MISC level, suggesting that these features were less tightly coupled to symptom progression.

**Figure 6:**
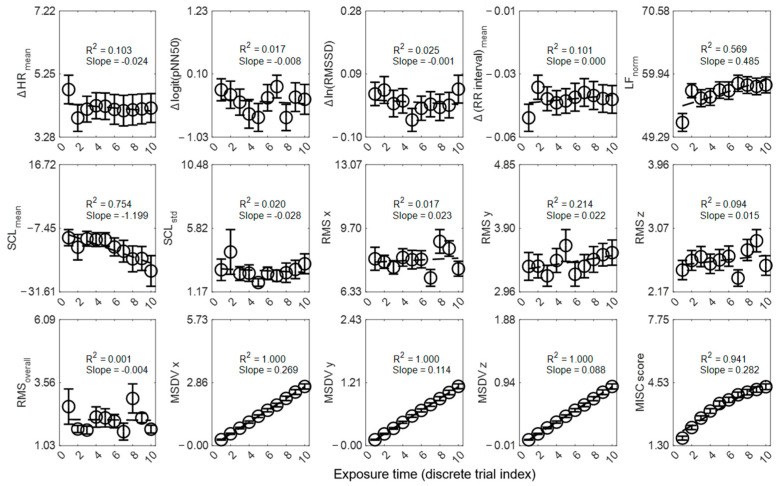
Exposure-time averaged feature trends across consecutive exposure intervals. Feature values were averaged within each of the ten 2 min intervals across participants, and error bars indicate standard error. Linear regression lines are shown for each feature, with regression slope and coefficient of determination reported in each panel.

The exposure-time averaging analysis revealed distinct associations across the exposure-time-averaged feature values (Figure 6). Specifically, all three axis-specific MSDV values showed perfect alignment with exposure time (R^2^ = 1.00). In the analysis, MSDV was treated as a cumulative quantity, accumulated across successive trials as a running sum of per-trial increments. Because we used fixed road profiles and constant trial duration, each trial contributed an equal MSDV increment, yielding a linear relationship between cumulative MSDV and trial index, and directly explains the observed R^2^ = 1.000 in Figure 6. Also, MISC score showed a strong correlation with exposure time (R^2^ = 0.941), consistent with a cumulative dose-response pattern of motion sickness under sustained vibration. LF_norm_ and SCL_mean_ showed moderate correlations (R^2^ = 0.569 and 0.754), whereas HR_mean_, RMS measures, and time-domain HRV indices showed weak or negligible temporal trends (R^2^ < 0.25).

### 3.4. Model Performance Across Regression and Classification Tasks

Table 4 summarizes the classification performance across the three dataset configurations and classification algorithms. The exposure time only (type 1) dataset achieved classification accuracies of 65–66% across SVC, random forest, and XGBoost models, with AUC values of 69–71% and F1 scores between 62 and 63%. The physiological and vibration-derived only (type 2) dataset achieved a modest improvement over the first dataset configuration, with accuracies ranging between 67 and 70%, and F1 scores of 65–69%. However, the combined (type 3) dataset achieved the highest performance across all classification models and all metrics, with accuracies between 76 and 77%, AUC scores of 82–84%, and an F1 score between 73 and 75%. The improvement of up to 6–9% across all algorithms after adding exposure time to the physiological and vibration-derived features demonstrates the added predictive value of exposure time. These results indicate that temporal exposure progression carries predictive information that autonomic and kinematic features alone do not fully capture.

Compared to existing multimodal motion sickness studies, the accuracy of 77% achieved by the combined dataset in our study is qualitatively in the range of better-performing multimodal approaches reported under controlled conditions. Li et al. [28] reported 68.9% accuracy using a multilayer perceptron with absolute EEG power spectral density, and Recenti et al. [29] reported 74.7% accuracy with random forest using EEG, EMG, and heart rate signals. It should be noted, however, that both studies investigated visually induced motion sickness (VIMS) in screen- or VR-based setups, which involve a fundamentally different induction mechanism from the whole-body vibration paradigm in our study, differing in stimulus type, neural pathways engaged, and symptom labeling schemes. Therefore, this comparison should be interpreted as a broad cross-domain reference rather than a direct equivalence. Within this limitation, our study using ECG, GSR, head acceleration, and exposure time achieved competitive performance relative to models that additionally used EEG or EMG.

Results from the regression models, summarized in Table 5, supported the same overall pattern observed in classification. The type 1 dataset remained around R^2^ ≈ 0.20 across SVR, Random Forest, and XGBoost. The type 2 dataset improved slightly, with R^2^ values of 0.207–0.295, whereas the type 3 dataset produced the strongest results, reaching R^2^ = 0.392–0.487, with XGBoost achieving the highest explanatory power. In addition, the combined dataset consistently yielded the lowest MSE and RMSE across all regression models.

Under LOSO cross-validation, the type 3 dataset achieved accuracies of 65.6–72.7% and AUC values of 0.763–0.837 across the three classifiers, with random forest performing best (accuracy 72.7 ± 9.3%, AUC 0.837 ± 0.090, F1 0.687 ± 0.155) as shown in Table 6. The type 2 dataset achieved accuracies of 57.3–61.0% and AUC values of 0.652–0.703. Compared to the trial-level cross-validation results, the type 3 dataset showed a performance reduction of approximately 4–6 percentage points in accuracy, indicating a modest drop from trial-level estimates. Overall, these results show that adding exposure time to the combined dataset explained motion sickness severity better than using physiological and vibration-derived features alone.

### 3.5. Permutation Importance and Feature Contribution Patterns

**Figure 7:**
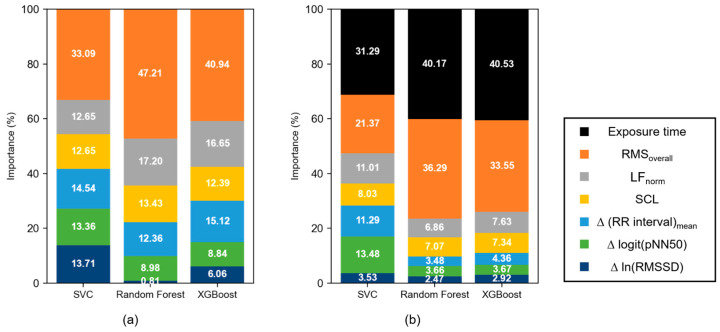
Permutation-based feature importance for motion sickness classification. Stacked bar charts show normalized importance percentages for each model. (**a**) Physiological and vibration-derived feature dataset, with RMS_overall_ ranked highest across SVC, Random Forest, and XGBoost. (**b**) Combined dataset, with exposure time ranked highest and RMS_overall_ ranked second across all models.

Figure 7 shows the normalized permutation importance results for the physiological and vibration-derived (type 2) dataset configuration (Figure 7a) and the combined (type 3) dataset configuration (Figure 7b). In the type 2 dataset, RMS_overall_ was the top-ranked feature across all three classification algorithms (SVC: 33.1%, random forest: 47.2%, XGBoost: 40.9%), while the other features contributed at broadly similar levels. This result suggests that vibration magnitude alone carried substantial predictive value even without temporal information. However, once exposure time was added, its feature importance exceeded that of all other variables across all classification algorithms (SVC: 31.3%, random forest: 40.2%, XGBoost: 40.5%), with RMS_overall_ consistently ranked second (SVC: 21.4%, random forest: 36.3%, XGBoost: 33.6%). The RMSSD showed the lowest importance across all models (2.9–3.7%), consistent with its failure to show significant statistical group differences or coherent severity trends. Also, LF_norm_, ∆(RR interval)_mean_, mean SCL, and pNN50 contributed at intermediate levels across the algorithms.

Trial-level feature contribution patterns were broadly consistent across all classification algorithms. In particular, the ranking order of exposure time > RMS_overall_ > physiological features were preserved across SVC, random forest, and XGBoost despite their different inductive mechanisms. This cross-algorithm agreement further supports the conclusion that exposure time and RMS_overall_ are the most dominant predictors of motion sickness in the classification models. Random forest and XGBoost produced closely matched feature-importance distributions, whereas SVC produced more uniform distributions across features. This pattern is consistent with the global margin-based structure of SVMs, in which individual feature contributions are not isolated as directly as in tree-based methods. Nonetheless, the feature-ranking order of exposure time and RMS_overall_ above the physiological features was consistent across all three models.

Notably, feature importance under LOSO remained consistent with the trial-level findings for the two dominant features, with exposure time (discrete trial index) and RMS_overall_ retaining their dominance across all models (Table 6). However, the remaining physiological features (LF, SCL, RR interval, RMSSD, pNN50) showed near-zero or negative importance under LOSO in both dataset configurations, indicating that their contribution under trial-level cross-validation was largely attributable to individual-specific physiological patterns rather than generalizable motion sickness signatures. Overall, the feature-importance results indicate that motion sickness prediction in this protocol is driven primarily by exposure progression and vibration burden, with ECG and GSR providing supporting but secondary information.

## 4. Discussion

### 4.1. Exposure Time Dominance and the Cumulative Dose-Response Interpretation

The central finding of this study is that exposure time, operationalized as a discrete trial index (1–10), was the most influential predictor of motion sickness across all classifiers and dataset configurations, and that adding it to the physiological and vibration-derived feature set consistently improved both classification and regression performance. The dominance of exposure time is consistent with the cumulative nature of motion sickness. Under repeated vibration exposure, symptoms do not appear abruptly in isolation; instead, they build gradually as sensory conflict accumulates. Therefore, exposure time, as operationalized in this study, functions as a compact proxy for temporal progression and symptom buildup. This finding is particularly consistent with sensory conflict theory, which describes motion sickness as the consequence of sustained conflict accumulation that exceeds an individual’s adaptive tolerance [1,2]. However, the observed dominance of exposure time should be interpreted as the dominance of cumulative exposure order under the controlled fixed vibration profiles used in this study, rather than as a direct replacement for MSDV in variable real-world driving environments.

The exposure-time-averaged MISC trend reflects this cumulative, threshold-like progression, in which symptoms increase across successive trial intervals rather than resetting between trials. Exposure time fills a gap that autonomic responses alone cannot address, because it explicitly represents how long participants have been exposed to motion. This helps explain why adding exposure time improved both classification and regression when combined with physiological and vibration-derived features. In short-to-moderate exposure scenarios, temporal information should therefore be treated as a primary feature rather than a nuisance variable, and real-time monitoring systems should explicitly include exposure history whenever it is available. However, in real vehicle environments where vibration magnitude fluctuates, road profiles change, or passengers take breaks, the dominance of exposure time may be reduced, and the independent contribution of physiological features may become more prominent. Also, MSDV remains the more physically meaningful cumulative vibration metric for real-world applications, whereas trial index was used in this study as a controlled experimental surrogate for exposure order.

### 4.2. Interpretation of Biosensor Feature Contributions

RMS_overall_ was the second most important predictor of motion sickness in this experimental protocol. This feature captures the role of head acceleration in motion sickness prediction. Larger values correspond to stronger whole-body vibration and reduced postural stability, both of which are relevant to the development and severity of motion sickness symptoms, as reported previously in [6]. The high feature importance and effect sizes observed for RMS_overall_ can also be linked to the translational acceleration used in this study and to the ISO 2631-1 frequency-weighting function, which emphasizes the 0.1–0.5 Hz range associated with peak human vestibular sensitivity.

Among the ECG features, LF_norm_ showed the highest permutation importance and the strongest association with motion sickness severity (d = 0.42, R^2^ = 0.807 in the severity-averaged analysis). Its increase with MISC severity is consistent with sympathetic activation accompanying nausea onset, including elevated heart rate, increased vasomotor activity, and reduced parasympathetic tone [8,30]. In addition, the moderate correlation between LF_norm_ and exposure time (R^2^ = 0.569) suggests that it captures both temporal accumulation and severity-related changes, making it distinct from the other ECG features. By contrast, RMSSD and pNN50 showed limited importance, which may reflect their lower sensitivity to autonomic changes during the 2 min trial structure used in this study.

Although SCL_mean_ showed limited feature importance, it exhibited an interesting physiological pattern. It increased with MISC severity in the severity-averaged analysis (R^2^ = 0.161) but decreased strongly with exposure time (R^2^ = 0.754). This opposing pattern is consistent with an initial sympathetic arousal response at motion sickness onset followed by attenuation as continued exposure produces habituation of the sympathetic response. Electrodermal habituation is well established in psychophysiological research and has also been reported in the context of motion sickness [9].

### 4.3. Practical Implications of Findings and Feature Selection

This study disentangles the relative roles of temporal exposure progression, physiological responses, and vibration-based features in motion sickness prediction. Through severity analysis, group-level comparisons, and feature importance analysis, we found that different feature families encode distinct aspects of motion sickness. Exposure time and MSDV capture the temporal accumulation of external motion burden. RMS_overall_ characterizes the body’s response to motion perturbation and is closely linked to the development of motion sickness symptoms. LF_norm_ and selected ECG/GSR features track motion sickness severity by reflecting internal physiological responses rather than exposure accumulation. However, the best model performance was obtained when all of these feature families were combined, indicating that motion sickness prediction benefits from integrating cumulative exposure, motion-exposure indicators, and autonomic responses.

Exposure time provided the strongest predictive gain across all analysis configurations. Therefore, in a feature-selection strategy, cumulative exposure time should be the first priority before adding biosensor-derived features. The next priority is RMS_overall_, which serves as a robust vibration summary measure. It is simple to compute and sensor-light, and it captures much of the relevant biomechanical information across axis-specific measures. ECG and GSR can then be added to provide complementary autonomic information beyond temporal accumulation and kinematics, particularly when sympathetic activation has not yet fully stabilized.

The LOSO cross-validation results provided some additional important insights. Substantively, the feature importance hierarchy shifted under LOSO in a revealing way, with exposure time and RMS_overall_ retaining their feature importance dominance, while ECG- and GSR-derived features dropped to near-zero importance. The drop in importance of the physiological signals suggests that autonomic physiological responses capture individual-specific rather than generalizable motion sickness signatures. Hence, deployable systems for unseen users may achieve adequate performance using vibration-derived features and cumulative exposure information only. This tiered sensing strategy is particularly useful for real-world system design, where performance, complexity, and cost must be balanced. It also suggests that future motion sickness prediction systems should not assume that more sensors automatically produce better models; instead, the most informative and stable features should be prioritized.

## 5. Limitations and Future Work

Despite the contributions of this study, several limitations should be acknowledged. First, the experiment was conducted under controlled motion-simulator conditions, so the findings may not fully generalize to real vehicle travel. Under real driving conditions, road profiles vary, vehicle speed changes, and passengers may take breaks or change posture, all of which could disrupt the monotonic accumulation of MSDV over time. As a result, the predictive value of exposure time (discrete trial index) and MSDV may differ in such environments, and the relative value of ECG and GSR may also increase or change. Therefore, validation in real vehicle environments and under longer exposure durations is required. Second, although baseline normalization reduced inter-subject variability, physiological responses remained heterogeneous across participants. Furthermore, the trial-level cross-validation strategy gave optimistic performance estimates, which became lower under LOSO evaluation. Nevertheless, the LOSO estimates remained modestly optimistic, and, notably, the feature importance dominance of exposure time and RMS_overall_ was preserved under subject-level evaluation. However, the collapse of physiological feature importance under LOSO suggests that ECG and GSR contributions are largely individual-specific; future work should explore subject-specific calibration or personalized baseline models to recover their generalizability. Third, the participant sample was limited to young adults from a relatively narrow demographic range. Because motion sickness susceptibility varies with age and other individual factors, future studies should include a broader age range and more diverse participants to improve generalizability.

Future investigations may also benefit from expanding sensing beyond physiological and acceleration-based measures. In real vehicle environments, occupants often change behavior as motion sickness severity increases. They may stop the ongoing activity and adopt mitigation behaviors such as closing their eyes or looking at a distant external target. Adding camera-based monitoring of head pose, gaze direction, and task interruption events may therefore provide complementary information for motion sickness prediction. Integrating such behavioral cues with physiological features would also allow future studies to assess the contribution of mitigation-related behaviors to prediction performance.

## Figures and Tables

**Table 1:** Symptom-based criteria for the misery scale (MISC). Each score (0–10) corresponds to an incremental severity level of motion sickness, ranging from no symptoms to vomiting.

Symptoms		Score
No problems		0
Uneasiness (no typical symptoms)		1
Dizziness, warmth, headache, stomach awareness, sweating, …	Vague	2
	Slight	3
	Fairly	4
	Severe	5
Nausea	Slight	6
	Fairly	7
	Severe	8
	Near retching	9
Vomiting		10

**Table 2:** Distribution of responses across MISC scores and their categorical grouping.

MISC Score	Number of Responses	Category
0	78	Weak motion sickness
1	158	
2	275	
3	366	
4	333	Strong motion sickness
5	128	
6	133	
7	54	
8	25	

**Table 3:** Group comparison between weak and strong motion sickness (MS) conditions across features. Values are reported as mean ± SE. Statistical differences were evaluated using Welch’s *t*-test, with corresponding effect sizes (Cohen’s d) and explained variance (R^2^). Significance levels are indicated as *p* < 0.05 *, *p* < 0.01 **, *p* < 0.001 ***, n.s.: not significant.

Type	Feature	Weak MS	Strong MS	*p*-Value	Cohen’s d	*R* ^2^	Significant
ECG	∆HR_mean_	3.68 ± 0.19	4.89 ± 0.21	<0.001	0.22	0.009	***
	∆logit(pNN50)	−0.34 ± 0.10	−0.47 ± 0.11	0.4	0.04	0.008	n.s.
	∆ln(RMSSD)	0.01 ± 0.02	0.00 ± 0.02	0.7	0.02	0.011	n.s.
	∆(RR_interval_)_mean_	−0.03 ± 0.00	−0.05 ± 0.00	<0.001	0.27	0.010	***
	LF_norm_	53.67 ± 0.59	60.75 ± 0.62	<0.001	0.42	0.031	***
GSR	SCL_mean_	−15.77 ± 1.84	−14.63 ± 1.69	0.5	0.02	0.023	n.s.
	SCL_std_	2.28 ± 0.38	2.28 ± 0.25	0.077	0.08	0.011	n.s.
Acceleration	RMS_x_	6.84 ± 0.19	9.55 ± 0.22	<0.001	0.48	0.049	***
	RMS_y_	2.87 ± 0.07	4.09 ± 0.09	<0.001	0.54	0.053	***
	RMS_z_	2.26 ± 0.06	3.09 ± 0.06	<0.001	0.49	0.052	***
	RMS_overall_	1.69 ± 0.18	2.56 ± 0.14	<0.001	0.19	0.089	***
	MSDV_x_	1.00 ± 0.03	2.08 ± 0.04	<0.001	1.00	0.220	***
	MSDV_y_	0.41 ± 0.01	0.89 ± 0.02	<0.001	1.10	0.271	***
	MSDV_z_	0.33 ± 0.01	0.67 ± 0.01	<0.001	0.99	0.211	***
Exposure time		4.56 ± 0.10	6.72 ± 0.09	<0.001	0.81	0.141	***

**Table 4:** Classification performance of SVC, Random Forest, and XGBoost across three dataset configurations: exposure time only (type 1), physiological and vibration-derived features only (type 2), and combined (type 3) dataset. Reported metrics include mean ± standard deviation of accuracy, AUC, precision, sensitivity, specificity, and F1 score obtained from cross-validation.

Classification Model	Dataset Configuration	Accuracy (%)	AUC (%)	Precision (%)	Sensitivity (%)	Specificity (%)	F1 Score (%)
SVC	Type 1	64 ± 4	69 ± 5	59 ± 4	68 ± 5	64 ± 6	63 ± 4
	Type 2	67 ± 3	74 ± 4	61± 4	69 ± 3	67 ± 5	65 ± 3
	Type 3	76 ± 3	82 ± 3	70 ± 4	76 ± 4	75 ± 4	73 ± 4
Random forest	Type 1	65 ± 4	71 ± 5	59 ± 5	65 ± 8	65 ± 6	62 ± 8
	Type 2	70 ± 4	76 ± 3	63 ± 4	75 ± 3	66 ± 5	69 ± 3
	Type 3	77 ± 3	84 ± 3	72 ± 4	78 ± 4	77 ± 3	75 ± 4
XGBoost	Type 1	65 ± 4	71 ± 5	59 ± 5	65 ± 7	65 ± 6	62 ± 5
	Type 2	70 ± 4	76 ± 4	64 ± 5	72 ± 5	69 ± 5	68 ± 5
	Type 3	77 ± 4	83 ± 4	72 ± 5	77 ± 5	76 ± 5	74 ± 5

**Table 5:** Regression performance comparison across three dataset configurations: exposure time only (type 1), physiological and vibration-derived features only (type 2), and combined (type 3) dataset for SVR, random forest, and XGBoost models. Values represent mean ± standard deviation of the coefficient of determination (R^2^), mean squared error (MSE), and root mean squared error (RMSE) obtained from cross-validation.

Regression Model	Dataset Configuration	R^2^	MSE	RMSE
SVR	Type 1	0.205 ± 0.034	2.552 ± 0.113	1.597 ± 0.036
	Type 2	0.207 ± 0.081	2.518 ± 0.301	1.584 ± 0.095
	Type 3	0.392 ± 0.062	1.929 ± 0.225	1.387 ± 0.081
Random forest	Type 1	0.196 ± 0.053	2.555 ± 0.247	1.597 ± 0.076
	Type 2	0.295 ± 0.049	2.246 ± 0.243	1.496 ± 0.083
	Type 3	0.457 ± 0.042	1.728 ± 0.193	1.312 ± 0.074
XGBoost	Type 1	0.196 ± 0.054	2.555 ± 0.248	1.597 ± 0.077
	Type 2	0.284 ± 0.058	2.275 ± 0.224	1.507 ± 0.076
	Type 3	0.487 ± 0.049	1.629 ± 0.160	1.275 ± 0.063

**Table 6:** Classification performance and dominant feature permutation importance under leave-one-subject-out (LOSO) cross-validation for physiological and vibration-derived features only (type 2) and combined (type 3) dataset configurations. Mean permutation importance values (F1-based) are shown in parentheses. All remaining physiological features (LFnorm in Type 3, SCL, RR interval, RMSSD, pNN50) showed near-zero or negative importance under LOSO, indicating limited generalizability to unseen participants. Bold indicates best overall performance.

Classification Model	Dataset Configuration	Accuracy (%)	AUC (%)	F1 Score (%)	Most Important Feature	2nd Most Important Feature
SVC	Type 2	0.573 ± 0.136	0.652 ± 0.146	0.488 ± 0.194	RMS_overall_ (0.081)	LF_norm_ (0.007)
	Type 3	0.656 ± 0.121	0.763 ± 0.117	0.587 ± 0.175	Exposure time (0.127)	RMS_overall_ (0.053)
Random forest	Type 2	0.610 ± 0.107	0.703 ± 0.110	0.574 ± 0.187	RMS_overall_ (0.099)	LF_norm_ (0.018)
	Type 3	**0.727 ± 0.093**	**0.837 ± 0.090**	**0.687 ± 0.155**	Exposure time (0.148)	RMS_overall_ (0.118)
XGBoost	Type 2	0.601 ± 0.133	0.674 ± 0.128	0.546 ± 0.181	RMS_overall_ (0.101)	LF_norm_ (0.038)
	Type 3	0.706 ± 0.090	0.814 ± 0.101	0.643 ± 0.166	Exposure time (0.141)	RMS_overall_ (0.100)

## Data Availability

Data will be made available on request.
